# How Reliable Is the G41 Discharge Code for Status Epilepticus?

**DOI:** 10.1002/brb3.70443

**Published:** 2025-03-18

**Authors:** Quentin Calonge, Vincent Navarro, Sophie Tezenas du Montcel

**Affiliations:** ^1^ Sorbonne Université, Paris Brain Institute‐Institut du Cerveau ICM, INSERM, CNRS, Pitié‐Salpêtrière Hospital Paris France; ^2^ AP‐HP, Epilepsy Unit, Pitié‐Salpêtrière Hospital, DMU Neurosciences Paris France; ^3^ AP‐HP, Center of Reference for Rare Epilepsies ERN EpiCARE, Pitié‐Salpêtrière Hospital Paris France; ^4^ Sorbonne Université, Institut du Cerveau, Paris Brain Institute ICM, CNRS, Inria, Inserm, AP HP Paris France

**Keywords:** medico‐administrative databases, positive predictive value, status epilepticus

## Abstract

**Introduction:**

Medico‐administrative databases are increasingly used to study the epidemiology of status epilepticus (SE), targeting hospitalizations with the SE G41 ICD‐10 code. However, the positive predictive value (PPV) of the G41 code, which measures the percentage of true cases among those identified by the code, is unknown.

**Methods:**

We identified all hospitalizations with a primary or secondary diagnosis coded as G41 in five different hospitals. Medical reports for each hospitalization were reviewed to classify the stays as really related to SE or not, using two distinct approaches (sensitive and specific). The clinical characteristics of SE cases were also extracted.

**Results:**

Among the 797 hospitalizations identified, the PPV ranged from 85.7% using the sensitive approach to 70.6% with the specific approach. Hospitalizations coded with G41 as the main diagnosis had the highest PPV, whereas codes G411 and G418 showed the lowest PPV. Of the 400 hospitalizations with a G410 (generalized convulsive SE) code, 72.7% were classified as generalized convulsive SE, while 76.5% of the 149 hospitalizations with a G412 (focal SE) code were classified as focal SE.

**Conclusion:**

Our findings highlight that PPV varies by G41 subtype and diagnostic position. Studies requiring a higher PPV should exclude certain codes or hospitalizations with G41 code only as an associated diagnosis. Further studies are needed to estimate the sensitivity and specificity of G41 code.

## Graphical abstract

Sorbonne University Hospitals (5 hospitals, 1.1% of hospitalizations in France Extraction of all hospitalizations with a G41 discharge code (n = 797). Review of medical reports and labeling of hospitalizations using a sensitive and a specific approach. True positive (TP): hospitalization with a G41 code that corresponds to a hospitalization for Status Epilepticus. False positive (FP): hospitalization with a G41 code without Status Epilepticus Positive Predictive Value (PPV) : TP / (TP + FP).

The Positive Predictive Value (PPV) of the G41 discharge code for Status Epilepticus ranges from 70.6% to 85.7%. The PPV is higher when the G41 code is the main diagnosis or when the patient is hospitalized in a neurology unit.

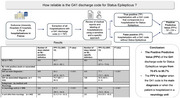

## 1 Introduction

Status epilepticus (SE) is a condition resulting either from the failure of the mechanisms responsible for seizure termination or from the initiation of mechanisms, which lead to abnormally, prolonged seizure, as defined in 2015 by the International League Against Epilepsy (ILAE). An operational definition considers the duration (t1) beyond which a seizure is unlikely to stop spontaneously. This duration was set at 5 min for generalized tonic–clonic seizures, 10 min for focal SE with impaired consciousness, and 10–15 min for absence SE (Trinka et al. 2015).

SE is a severe disorder with an incidence of 9.9 to 43/100,000 person‐years (Sánchez and Rincon 2016) and mortality rates up to 40% (Sutter and Kaplan 2013). Medico‐administrative databases provide valuable information for understanding SE epidemiology, and an increasing number of studies was conducted on hospitalizations identified by the International Classification of Diseases‐10 (ICD‐10) code G41 for SE (Choi et al. 2022; Guterman et al. 2021; Mevius et al. 2023; Ong et al. 2015; Calonge et al. 2024). Determining the positive predictive value (PPV) of the G41 code for identifying cases of SE is essential for interpreting the results of these studies, as it provides information on how many hospitalizations coded as G41 truly correspond to SE. Several studies have suggested that the PPV of the G41 code is high (87.3% in a study of 118 patients hospitalized in neurology units (Zelano et al. 2014), and up to 100% for the G41.0 code in another study involving patients hospitalized or treated in emergency departments (Jetté et al. 2010)). However, these studies were limited to patients hospitalized in neurology units or involved small patient samples with a G41 code. Our study aimed to evaluate the PPV of G41 code for SE in a large sample of patients hospitalized across various medical units.

## 2 Methods

### 2.1 Study Design and Data Source

We conducted a retrospective study using hospitalization data from 2019 and 2022 from five hospitals of the “Assistance Publique—Hôpitaux de Paris” (AP‐HP) affiliated with Sorbonne Université, representing 1.1% of total hospitalizations in France in 2022 (number extracted from the French health insurance database).

For each hospitalization, *main diagnosis* (MD) and additional *associated diagnoses* (AD), for complications or comorbidities requiring extra management, are provided with ICD‐10 diagnostic codes. In France, coding is performed in each unit by the senior physician who takes care of the patient and is subsequently validated by an expert (physician or technician) in coding. This study was approved by an internal review board, and approval for data reuse was obtained from the heads of medical units. This study was reported according to the STROBE statement.

### 2.2 Study Population

We included all hospitalizations from the five targeted hospitals where an SE G41 code appeared as the MD or AD. Patients could be included several times in case of multiple hospitalizations. Exclusion criteria included outpatient hospitalizations and those lacking medical reports.

### 2.3 SE Labeling

Each medical chart was reviewed by an epileptologist (Q.C.). All available information were used for the labeling process: hospitalization reports and, when available, EEG and brain imaging reports. SE was defined as a generalized convulsive seizure persisting beyond 5 min or a focal seizure with impaired consciousness or absence persisting beyond 10 min, by the ILAE (Trinka et al. 2015). For seizures for which the temporal limit was not explicitly defined by the ILAE (e.g., myoclonic SE), we used the 10‐min threshold to define the SE. However, due to occasional incomplete reporting by clinicians (e.g., duration of symptoms, recovery of consciousness between seizures), we adopted two labeling approaches:

– A “sensitive” approach (sensitive definition), where we labeled as SE any hospitalization in which the clinician suggested SE with compatible semiology, even if the duration was unclear, the lack of recovery between seizures was not explicitly mentioned, or the semiology was unprecise (e.g., impaired consciousness improving after antiepileptic treatment).
– A “specific” approach (specific definition), where SE was labeled only when the medical report explicitly stated that the seizure lasted more than 5 min for generalized convulsive seizures or more than 10 min for focal or other seizures.
– Cases in which seizures stopped spontaneously or after treatment administered within 5 min (for generalized convulsive seizures) or 10 min (for focal seizures) from symptom onset were not labeled as SE.

Finally, cases where the medical report did not mention SE or where a diagnosis of psychogenic non‐epileptic seizures (PNES) was established were not labeled as SE.

### 2.4 Statistical Analysis

We measured the PPV of diagnostic codes to identify SE according to the sensitive and specific definition and the position of the G41 code (MD or AD). Several subgroup analyses were conducted according to (i) the type of medical unit (intensive care unit [ICU] or neurology unit); (ii) age groups (< 18 years, 18–64 years, 65+ years); and (iii) the subtype of SE code (G41.0: generalized convulsive SE, G41.1: absence SE, G41.2: focal SE, G41.8: “other” SE such as myoclonic SE, G41.9: unspecified SE). We calculated 95% confidence intervals for each PPV and used a chi‐square test to compare proportions where appropriate, with an alpha threshold of 5% considered significant. All analyses were performed on R version 4.1.3.

We extracted the reported semiology for each SE episode, distinguishing several simplified categories: generalized convulsive, focal, other motor seizure (myoclonic, tonic, and hyperkinetic), and nonconvulsive seizure with impaired consciousness (absence). Certain G41 subtypes may help target specific semiological categories (e.g., G41.0 “Grand mal status epilepticus” for generalized convulsive SE). Table 1 presents the correspondence between the extracted semiology, the 2015 ILAE classification, and the most appropriate ICD‐10 code for identifying each type of SE.

**TABLE 1:** Correspondence between the extracted semiology, the 2015 ILAE classification, and the most appropriate ICD‐10 code.

Semiological classification	Classification of status epilepticus (SE) according to the ILAE 2015 classification	ICD‐10 code considered as appropriate for this type of SE
Generalized convulsive	A.1 Consulsive SE – A.1.a Generalized convulsive – A.1.b Focal onset evolving into bilateral convulsive SE – A.1.c Unknown whether focal or generalized	G410 (Grand mal SE)
Focal	A.3 Focal motor – A.3.a Repeated focal motor seizures (Jacksonian) – A.3.b Epilepsia partialis continua (EPC) – A.3.c Adversive status – A.3.d Oculoclonic status – A.3.e Ictal paresis	G418 (Other SE)
	B.2 NCSE without coma B.2.b Focal – B.2.b.a Without impairment of consciousness	G418 (Other SE)
	B.2 NCSE without coma B.2.b Focal – B.2.b.b Aphasic status	G418 (Other SE)
	B.2 NCSE without coma B.2.b Focal – B.2.b.c With impaired consciousness	G412 (Complex partial SE)
Other motor manifestation	A.2 Myoclonic SE (prominent epileptic myoclonic jerks) – A.2.a With coma – A.2.b Without coma A.4 Tonic status A.5 Hyperkinetic SE	G418 (Other SE)
Non convulsive with impaired consciousness	B.1 NCSE with coma (including so‐called “subtle” SE) B.2 NCSE without coma B.2.a Generalized – B.2.a.a Typical absence status – B.2.a.b Atypical absence status – B.2.a.c Myoclonic absence status	G411 (Petit mal SE)

## 3 Results

### 3.1 Population Characteristics

We identified 821 hospital stays across 50 departments where a G41 code was listed as MD or AD. We excluded 24 hospital stays due to outpatient stay (*n* = 11) or lack of a medical report (*n* = 15). A total of 797 hospital stays involving 690 unique patients were included in the main analysis. Among these stays, 379 (47.6%) involved female patients, and the median age was 49 years (interquartile range [IQR]: 16–67). We labeled 683 hospital stays as SE based on the sensitive definition and 563 based on the specific definition. Details of hospital stays characteristics are presented in Table 2.

**TABLE 2:** Population characteristics.

	All: *n* = 797 hospital stays
Female	379 (47.6%)
Age (years)	49 (16–67)
**Number of hospital stays labeled as SE**	
Sensitive definition	683 (85.6%)
Specific definition	563 (70.6%)
**Hospital stays not labeled as SE**	
Seizure without SE	59 (7.4%)
PNES	13 (1.6%)
Absence of seizure	41 (5.2%)
**Position of G41**	
Main diagnosis	564 (70.8%)
Associated diagnosis	233 (29.2%)
**Types of seizure**	
Generalized convulsive	443 (55.6%)
Focal	265 (33.2%)
Other Motor	19 (2.4%)
Absence	15 (1.9%)
Dystonic	1 (0.1%)

### 3.2 PPV of Different Variables

Overall, the PPV of the G41 code for SE was 85.7% according to the sensitive definition and 70.6% according to the specific definition. The PPV was higher when the G41 code was the MD (90.2% vs. 74.7% for G41 code as AD for sensitive definition, *p* < 0.001), and in the presence of a stay in a neurology unit (91.3% for hospital stays with neurology unit vs. 81.4% without, *p* < 0.001). There was no significant difference in PPV between hospital stays with or without ICU, and the PPV was slightly lower for the 18–64 age group using the specific definition of SE (66.8% vs. 76.3% for < 18 and 71.7% for 65+, *p* = 0.049, Tables 3, 4, 5). Subtypes of the G41 code showed PPVs ranging from 72.4% to 91.3% according to the sensitive definition, with G41.2 having the highest PPV and G41.8 the lowest (Table S1). For each code (except G41.1), the PPV was higher when the code was the MD rather than AD, with significant differences for G41.0 (92.1% vs. 76.1%, *p* < 0.001) and G41.9 (89.1% vs. 72.0%, *p* = 0.009).

**TABLE 3:** PPV of the G41 code for all hospitalizations and according to location of the code (main diagnosis or associated diagnosis).

	Number of stays labeled as SE: sensitive definition	PPV (%) (95% CI)	*p* value	Number of stays labeled as SE: specific definition	PPV (%) (95% CI)	*p* value
All (*n* = 797)	683	85.7 (83.3–88.1)		563	70.6 (67.5–73.8)	
**By code position (main or associated diagnosis)**						
G41 as main diagnosis: (*n* = 564)	509	90.2 (87.8–92.7)	**< 0.001**	435	77.1 (73.7–80.6)	**< 0.001**
G41 as associated diagnosis (*n* = 233)	174	74.7 (69.1–80.3)		128	54.9 (48.5–61.3)	

*Note*: The positive predictive values (the proportion of true positives among selected hospitalizations) were tested using a chi‐square test. The corresponding *p* values are reported in the table.

**TABLE 4:** PPV of the G41 code according to the medical unit (neurology and ICU).

	Number of stays labeled as SE: sensitive definition	PPV (%) (95% CI)	*p* value	Number of stays labeled as SE: specific definition	PPV (%) (95% CI)	*p* value
**Medical unit**						
**Neurology**						
In the neurology unit (*n* = 355)	324	91.3 (88.0–94.0)	**< 0.001**	282	79.4 (74.9–83.4)	**< 0.001**
In unit other than neurology (*n* = 442)	360	81.4 (77.8–85.1)		282	63.8 (59.3–68.3)	
**ICU**						
In ICU (*n* = 295)	258	87.5 (83.7–91.2)	0.32	211	71.5 (66.4–76.7)	0.73
In units other than ICU (*n* = 502)	425	84.7 (81.5–87.8)		352	70.1 (66.1–74.1)	

*Note*: The positive predictive values (the proportion of true positives among selected hospitalizations) were tested using a chi‐square test. The corresponding *p* values are reported in the table.

**TABLE 5:** PPV of the G41 code by age group.

	Number of stays labeled as SE: sensitive definition	PPV (%) (95% CI)	*p* value	Number of stays labeled as SE: specific definition	PPV (%) (95% CI)	*p* value
**Subgroups: by age group**						
< 18 (*n* = 207)	178	86.0 (81.3–90.7)	0.28	158	76.3 (70.5–82.1)	**0.049**
18–64 (*n* = 364)	305	83.8 (80.0–87.6)		243	66.8 (61.9–71.6)	
65+ (*n* = 226)	200	88.5 (84.3–92.7)		162	71.7 (65.8–77.6)	

*Note*: The positive predictive values (the proportion of true positives among selected hospitalizations) were tested using a chi‐square test. The corresponding *p* values are reported in the table.

### 3.3 Type of Seizure According to G41 Code

Among the 400 hospitalizations with a G41.0 code, 72.7% referred to generalized convulsive seizures, while 76.5% of the 149 hospitalizations with a G41.2 code referred to focal seizures. Hospitalizations with G41.1, G41.8, or G41.9 codes exhibited a variety of seizure types (Table 6).

**TABLE 6:** Type of seizure identified during hospitalization according to the presence of each type of G41.x code.

Code (number of hospital stays)	Generalized convulsive seizure	Focal seizure	Nonconsulsive seizure (absence)	Other motor seizure	PNES	Absence of seizure
G410 (*n* = 400)	291 (72.7%)	71 (17.7%)	5 (1.3%)	9 (2.3%)	9 (2.3%)	15 (3.7%)
G411 (*n* = 35)	8 (22.9%)	17 (48.5%)	3 (8.6%)	3 (8.6%)	2 (5.7%)	2 (5.7%)
G412 (*n* = 149)	25 (16.8%)	114 (76.5%)	3 (2.0%)	0	0	7 (4.7%)
G418 (*n* = 58)	27 (46.6%)	21 (36.2%)	1 (1.7%)	3 (5.2%)	1 (1.7%)	5 (8.6%)
G419 (*n* = 179)	104 (58.1%)	53 (29.6%)	3 (1.7%)	5 (2.8%)	1 (0.5%)	13 (7.3%)

## 4 Discussion

We conducted the first comprehensive assessment of the PPV of the G41 code to identify SE, considering both the code's position (MD or AD) and subtype (e.g., G41.0, G41.1), across all hospitalizations with a G41 code in five different hospitals over a 2‐year period. Overall, we found that the PPV for identifying SE ranged from 70.6% to 85.7%. Previous studies have evaluated the PPV of G40 and G41 codes to identify epilepsy, consistently highlighting their high PPV despite potential sensitivity limitations (Mbizvo et al. 2020). Some studies reported a higher PPV for G41 compared to G40 (Jetté et al. 2010; Mbizvo et al. 2020), although subtypes like G41.8 or G41.9 showed lower performance.

Our study emphasizes the impact of G41 code position on PPV, with higher PPV when listed as the MD compared to an AD. To our knowledge, no previous study has reported the PPV of G40 and G41 codes based on position, though this has been observed in other conditions, such as myocarditis, where MD codes show better PPV (Wu et al. 2023). The G41 code, as the main diagnosis, is likely coded with more care by healthcare professionals since it refers to the main reason for hospitalization and is mandatory, while the associated diagnosis is optional, and may be used when the certainty of SE is less clear. We also observed increased PPV to identify SE when coding originated from a neurology unit, aligning with findings from neurology and epileptology departments that showed the highest PPVs (Reid et al. 2012) to identify epilepsy, whereas coding from ICU stays did not show a similar effect.

Age influences the PPV of epilepsy‐targeting algorithms using ICD‐10 codes, with lower PPV reported in older patients (Moura et al. 2019). In our study, PPV for SE was lower in the 18–64 age group compared to younger patients, but only when applying the “specific” definition, which requires precise reporting of SE duration. This may reflect differences in reporting practices across age groups, as younger patients, often admitted to neuro‐pediatric units, may have better documentation of SE.

For G41.0 (generalized convulsive SE) and G41.2 (focal SE) codes, we only found 72.7% and 76.5% of generalized convulsive and focal seizures, respectively. These results are slightly lower than those reported for a combination of G40.X codes for identifying generalized or focal seizures (Smith et al. 2020). This may be due to a lack of interest in coding from neurologists, since codes G41.0 and G41.2 are equally valuable for the stay. Conversely, G41.8 and G41.9 codes are less valued, yet we identified a significant number of generalized convulsive or focal SE among these hospitalizations. This suggests that accurate coding could enhance the recognition of these cases, highlighting the need to educate clinicians on coding practices.

While our study benefits from a substantial sample of SE‐related hospitalizations (*n* = 797) across 50 departments, it has certain limitations. The reliance on a single neurologist (Q.C.) for report review and labeling may introduce bias, despite efforts to use explicit and reproducible criteria. In addition, our analysis on G41‐coded hospitalizations does not allow us to assess the sensitivity and specificity of the G41 code for identifying SE, as we lack access to hospitalizations for SE coded with other classifications (e.g., G40) or those where SE was not diagnosed. This is particularly relevant for nonconvulsive SE, which is challenging to diagnose and likely underreported (Leitinger et al. 2019). We also did not have access to SE with rapid recovery following prehospital management, although these patients are likely to be hospitalized for observation, as recommended by the French Society of Intensive Care in 2018 (Recommandations Formalisées d'Experts n.d.). Further studies are needed to better evaluate the sensitivity and specificity of the G41 code.

## 5 Conclusion

Overall, this study is the first to assess the PPV of the G41 code for identifying SE, revealing that PPV varies based on the specific G41 code and its position (MD or AD). Future studies requiring greater certainty regarding the presence of SE should exclude certain G41 codes (e.g., G41.8) or restrict the analysis to hospitalizations with the G41 code listed as the MD.

## Author Contributions

**Quentin Calonge**: conceptualization, investigation, formal analysis, writing – original draft preparation, writing – review and editing. **Vincent Navarro**: conceptualization, resources, writing – review and editing. **Sophie Tezenas du Montcel**: conceptualization, data curation, writing – review and editing.

## Disclosure

We confirm that the manuscript complies with all instructions to authors. We confirm that this manuscript has not been published elsewhere and is not under consideration by another journal.

## Ethics Statement

This study was approved by an internal review board and ethics committee, and approval for data reuse was obtained from the heads of medical units.

## Conflicts of Interest

Vincent Navarro reports personal fees from UCB Pharma, EISAI, GW Pharma, and Angelini, outside the submitted work. Quentin Calonge reports support for attending meeting (Journées françaises de l’épilepsie) from UCB Pharma in 2023 and 2024, the remaining authors have no conflicts of interest.

### Peer Review

The peer review history for this article is available at https://publons.com/publon/10.1002/brb3.70443

## Supporting information



Supporting Information

## Data Availability

The anonymized datasets generated and analyzed during the current study are available from the corresponding author upon reasonable request.
