# Supplementary material for: How Reliable Is the G41 Discharge Code for Status Epilepticus?
Source: Brain Behav. 2025 Mar 18;15(3):e70443. doi: 10.1002/brb3.70443 (PMC11919778; doi:10.1002/brb3.70443)
Supplement: Supplementary file 1 — Supporting Information [file BRB3-15-e70443-s001.docx]

**Supplementary Table 1**: **Positive Predictive Value of Each G41.x Code for Status Epilepticus Based on Code Position (Principal or Associated Diagnosis) and Labeling Criteria (Sensitive or Specific).** For each G41 code subtype, differences in positive predictive values (corresponding to the proportion of true positives out of all hospitalizations identified with this code) based on the position of the code (principal or associated diagnosis) were tested using the Chi-square test, and the P-values are reported in the respective columns.

**G410: Positive Predictive Value by code position**

| **Position of code (n = number of hospital stays)** | Number of stays labeled as SE : **sensitive definition** | PPV (%) (95% CI) | P value | Number of stays labeled as SE : **specific definition** | PPV (%) (95% CI) | P value |
| --- | --- | --- | --- | --- | --- | --- |
| All (n = 400) | 347 | 86.8 (83.4 – 90.1) |  | 282 | 70.5 (66.0 – 75.0) |  |
| Main Diagnosis (n = 266) | 255 | 92.1 (88.9 – 95.3) | **< 0.001** | 208 | 78.2 (73.2 – 83.2) | **< 0.001** |
| Associated Diagnosis (n = 134) | 102 | 76.1 (68.9 – 83.3) |  | 74 | 55.2 (46.8 – 63.6) |  |

**G411: Positive Predictive Value by code position**

| **Position of code (n = number of hospital stays)** | Number of stays labeled as SE : **sensitive definition** | PPV (%) (95% CI) | P value | Number of stays labeled as SE : **specific definition** | PPV (%) (95% CI) | P value |
| --- | --- | --- | --- | --- | --- | --- |
| All (n = 35) | 30 | 85.7 (74.1 – 97.3) |  | 23 | 65.7 (50.0 – 81.4) |  |
| Main Diagnosis (n = 20) | 15 | 75.0 (56.0 – 94.0) | 0.11 | 12 | 60.0 (38.5 – 81.5) | 0.64 |
| Associated Diagnosis (n = 15) | 15 | 100 (94.9 – 100.0) |  | 11 | 73.3 (50.5 – 96.1) |  |

**G412: Positive Predictive Value by code position**

| **Position of code (n = number of hospital stays)** | Number of stays labeled as SE : **sensitive definition** | PPV (%) (95% CI) | P value | Number of stays labeled as SE : **specific definition** | PPV (%) (95% CI) | P value |
| --- | --- | --- | --- | --- | --- | --- |
| All (n = 149) | 136 | 91.3 (86.7 – 95.8) |  | 117 | 78.5 (71.9 – 85.1) |  |
| Main Diagnosis (n = 103) | 97 | 94.2 (89.7 – 98.7) | 0.12 | 84 | 81.6 (74.1 – 89.0) | 0.26 |
| Associated Diagnosis (n = 46) | 39 | 84.8 (74.4 – 95.2) |  | 33 | 71.7 (58.7 – 84.8) |  |

**G418: Positive Predictive Value by code position**

| **Position of code (n = number of hospital stays)** | Number of stays labeled as SE : **sensitive definition** | PPV (%) (95% CI) | P value | Number of stays labeled as SE : **specific definition** | PPV (%) (95% CI) | P value |
| --- | --- | --- | --- | --- | --- | --- |
| All (n = 58) | 42 | 72.4 (60.9 – 83.9) |  | 31 | 53.4 (40.6 – 66.3) |  |
| Main Diagnosis (n = 34) | 25 | 73.5 (58.7 – 88.4) | 1 | 20 | 58.8 (42.3 – 75.4) | 0.48 |
| Associated Diagnosis (n = 24) | 17 | 70.8 (52.6 – 89.0) |  | 11 | 45.8 (25.9 – 65.8) |  |

**G419: Positive Predictive Value by code position**

| **Position of code (n = number of hospital stays)** | Number of stays labeled as SE : **sensitive definition** | PPV (%) (95% CI) | P value | Number of stays labeled as SE : **specific definition** | PPV (%) (95% CI) | P value |
| --- | --- | --- | --- | --- | --- | --- |
| All (n = 179) | 151 | 84.4 (79.0 – 89.7) |  | 131 | 73.2 (66.7 – 79.7) |  |
| Main Diagnosis (n = 129) | 115 | 89.1 (83.8 – 94.5) | **0.009** | 99 | 76.7 (69.5 – 84.0) | 0.12 |
| Associated Diagnosis (n = 50) | 36 | 72.0 (59.6 – 84.4) |  | 32 | 64.0 (50.7 – 77.3) |  |
